# Supplementary material for: Scalable room temperature incorporation of CO2-selective ångström-scale pores in graphene for carbon capture
Source: Nat Commun. 2025 Nov 24;16:10380. doi: 10.1038/s41467-025-65336-4 (PMC12644594; doi:10.1038/s41467-025-65336-4)
Supplement: Supplementary file 1 — Supplementary Information [file 41467_2025_65336_MOESM1_ESM.pdf]

Supplementary Information

Scalable room temperature incorporation of CO_2-selective pores in graphene for carbon capture

Laboratory of Advanced Separations (LAS), École Polytechnique Fédérale de Lausanne (EPFL), 1950 Sion, Switzerland

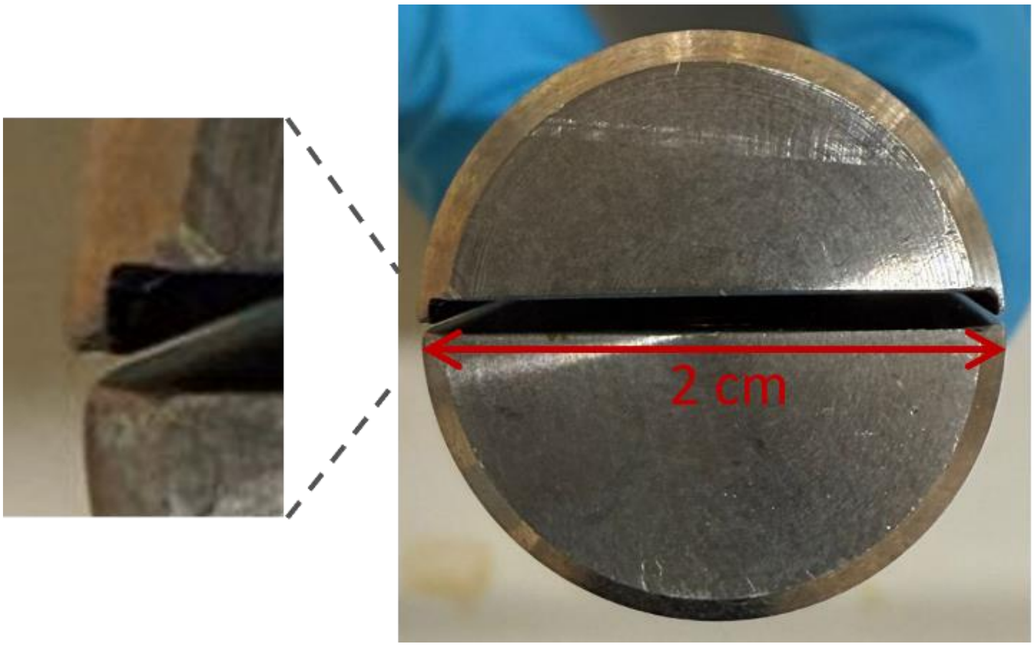

Figure S1. Side view of flow channel.

Note S1. Mechanical stability analysis of graphene samples inside slit flow reactor

The forces on the graphene coupon can be estimated using following equations;

$$F_{drag} = \frac{1}{2}\rho v^2 A C_d$$

where ρ is gas density ($\sim 1.3\ kg\,m^{-3}$ for O_2 at 25 ℃), A is the frontal area of Cu supported graphene sample exposed to flow (1.5 cm x 100 μm), C_d is drag coefficient (~1.2 for a flat plate in laminar flow, v is velocity of ozone (15-45 $cm\,s^{-1}$) near the graphene surface in the flow channel.

For 1.5 x 6 cm^2 graphene sample placed in FC300 reactor;

$$F_{drag} = \frac{1}{2}(1.3\ kg\,m^3) \times (0.45\ m\,s^{-1})^2 \times (1.5 \times 10^{-6} m^2) \times 1.2 = \sim 2.1\ x\ 10^{-7} N$$

Given that the mass (m) of a 1.5 x 6 cm^2 graphene coupon is 0.46 g,

$$F_{graviy} = mg = 0.46\ g \times 9.8\ m\,s^{-2} = \sim 4{,}5 \times 10^{-3} N$$

If we take into account the frictional force between Cu support and stainless steel with friction constant μ= 0.4 ;

$$F_{friction} = \mu \times F_{graviy} = \sim 1.8 \times 10^{-3} N$$

F_{drag} is almost negligible compared to frictional resistance, ensuring the graphene sample remains fixed under treatment conditions.

Note S2. Reynolds number, Peclet number, and boundary layer thickness estimation of different flow configurations

Assuming diluted inlet stream as 100 sccm pure O_2 at 25 C, 1bar; ρ is fluid density (1.3 kg/m^3), μ is the dynamic viscosity (2×10^{-5} Pa.s), and V is the average velocity (0.005, 0.09, 0.3 m/s for w/o flow channel, FC900 and FC300, respectively), H is hydraulic diameter (2, 0.172, and 0.059 cm, for w/o flow channel, FC900 and FC300, respectively), and D is diffusivity (2×10^{-5} m^2 s^{-1}), Reynolds and Peclet numbers can be calculated by following equations:

$$Re = \frac{\rho V H}{\mu}$$

$$Pe = \frac{VH}{D}$$

CFD simulations with velocity profiles of various flow configurations are shown below. Profile lines for each flow configuration are indicated with red dashed lines. Boundary layer thickness is estimated as the distance where velocity is reached to 99% of the maximum velocity in the reactor.

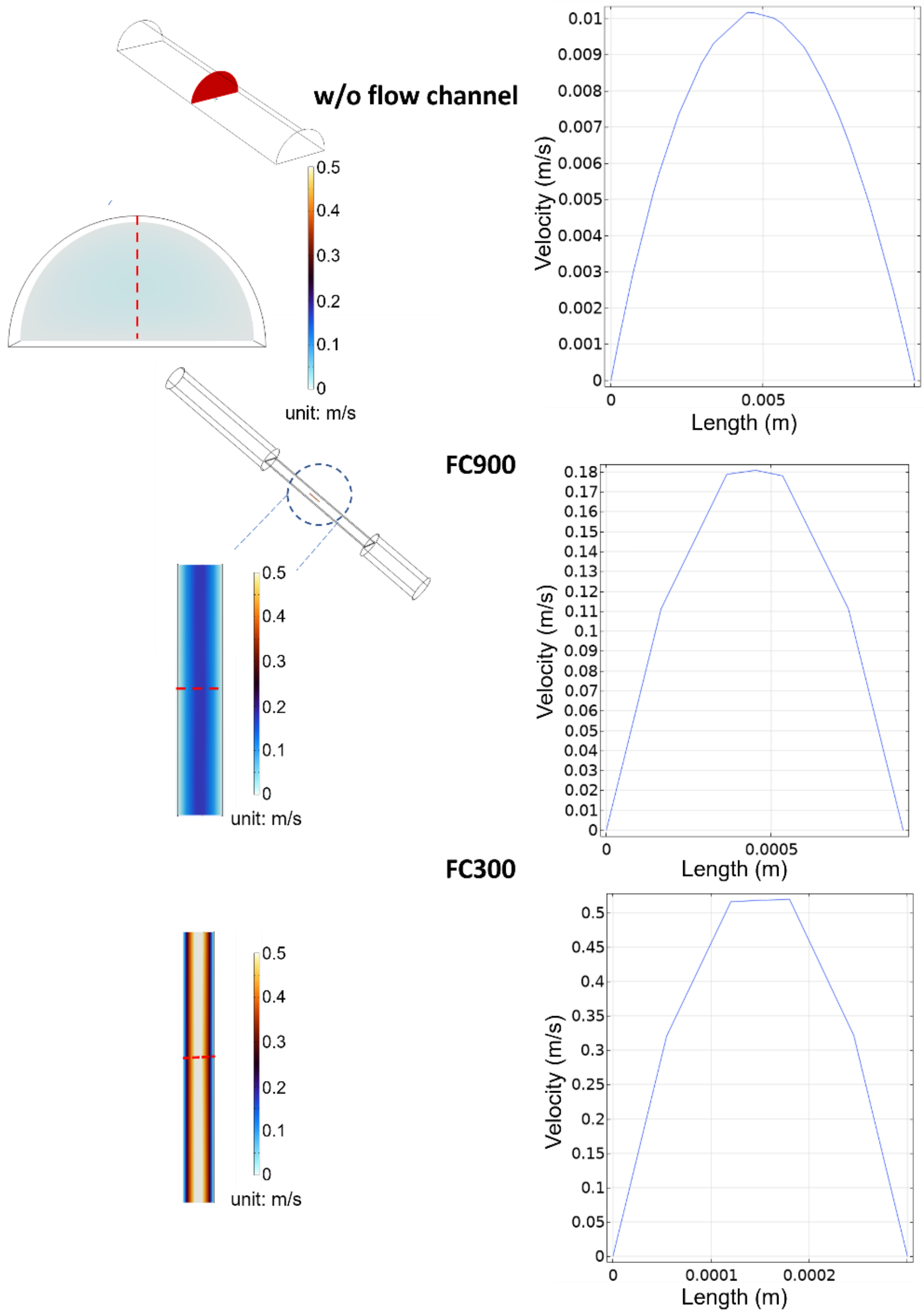

Figure S2. CFD simulations of different flow configurations showing velocity profiles. Source data are provided as a Source Data file.

Table S1. Reynolds number, Peclet number, and boundary layer thickness in various flow configurations.

**Flow Condition**	**Re**	**Pe**	**δ (mm)**
w/o Flow Channel	7	5	~ 4.3
FC900	10.7	90	~ 0.4
FC300	11.5	300	~ 0.12

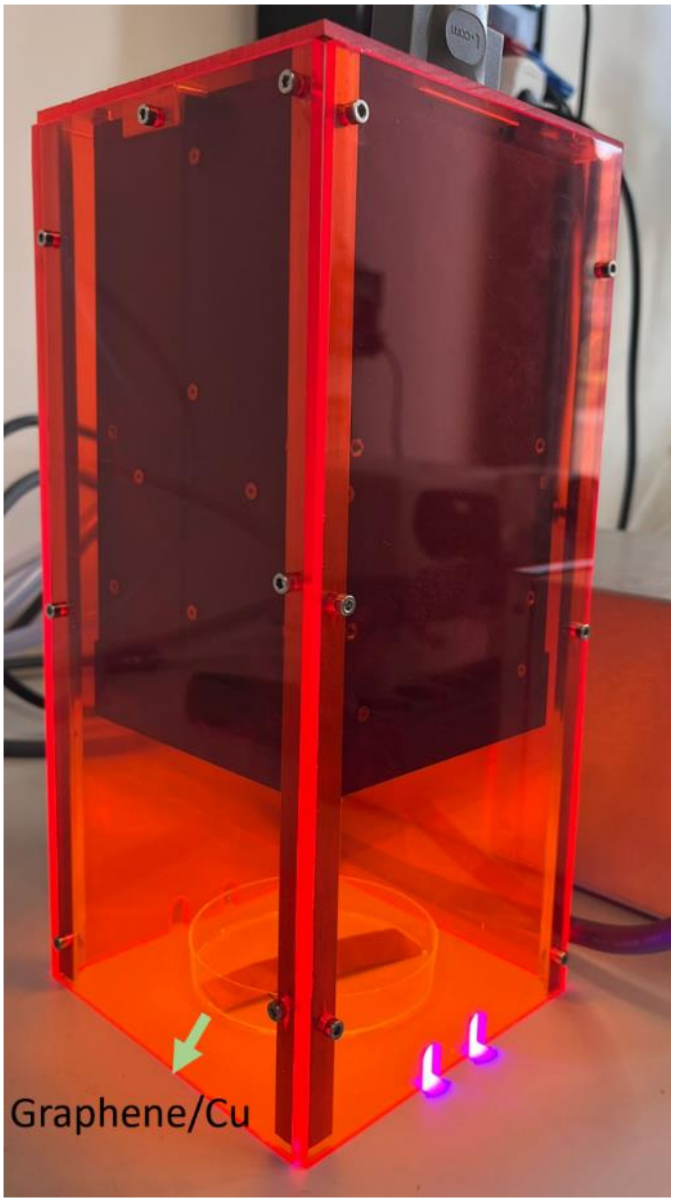

Figure S3. Custom made light (3.2 eV, 390 nm) used in photonic gasification

Note S3. Defect density and average distance between defects estimation based on Raman spectroscopy data

The change in defect density in oxidized graphene samples under different conditions can be evaluated using the relationship between Raman intensities and the average distance between defects, (L_D). For high-defect-density samples, the ratio I_D/I_G decreases with increasing porosity in the graphene lattice, indicating a reduction in the number of ordered six-atom ring.[1,2] For graphene samples exposed to O_3 without a flow channel, with FC900, and with FC300, the I_D/I_G ratio initially increased from 0.13 to 1.1 and then decreased to 0.8, respectively. This trend indicates that the samples were in a high-defect-density stage when oxidized with FC900 and FC300. Similarly, when samples were treated with a second cycle of O_3, the I_D/I_G ratio gradually decreased from 2.25 to 1, corresponding to exposure durations of 5 to 30 minutes, respectively. This also confirms that the samples were in a high-defect-density stage. In the low-defect-density stage (e.g., for the sample oxidized without a flow channel), the average distance between defects, L_D , can be calculated using the following correlation:[2]

$$L_D{}^2(nm^2) = (1.8 \pm 0.5) \times 10^{-9}\lambda_L^4\,(\frac{I_D}{I_G})^{-1}$$

Using the average defect distance L_D the defect density n_D can be estimated by:

$$n_D(cm^{-2}) = \frac{(1.8 \pm 0.5) \times 10^{22}}{\lambda_L^4}\left(\frac{I_D}{I_G}\right)$$

In high defect density stage, L_D and n_D can be calculated based on the following equations:

$$\left(\frac{I_D}{I_G}\right) = D(\lambda) \times\; L_D{}^2$$

where $\lambda = 457\ nm$, and $D(\lambda) = 0.039\ nm^{-2}$.[3]

$$n_D(cm^{-2}) = \frac{10^{14}}{\pi L_D^2}$$

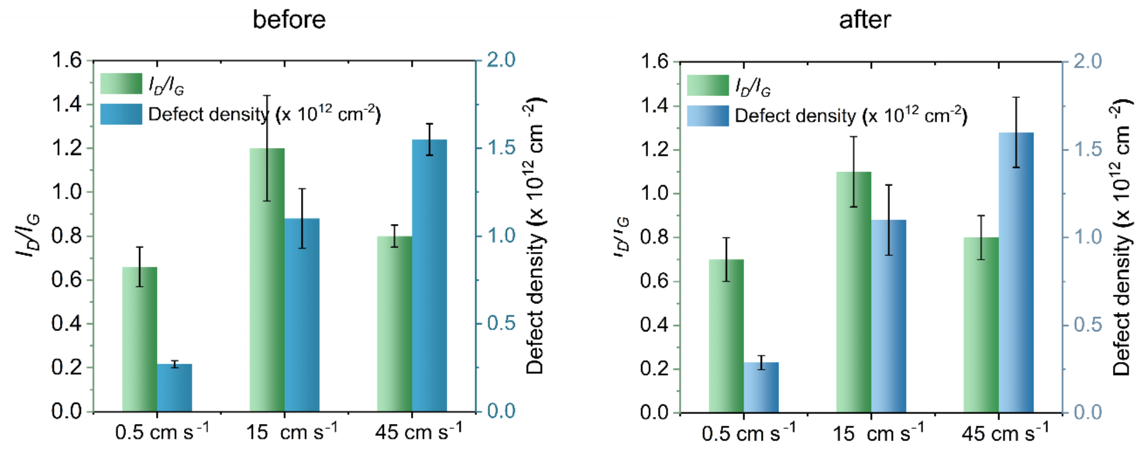

Figure S4. Quantification of evolution of the I_D/I_G ratio and defect density, analyzed based on the carbon amorphization trajectory, for the three velocities, before and after lattice gasification. Source data are provided as a Source Data file.

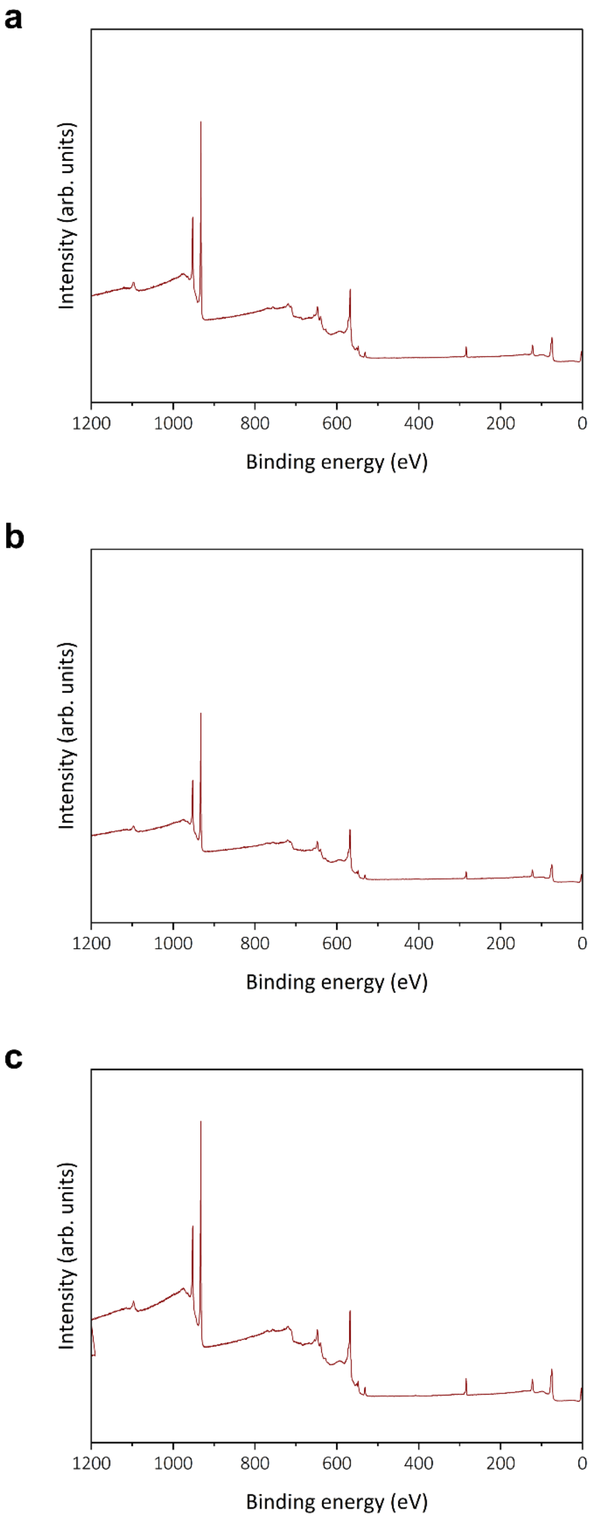

Figure S5. XPS wide spectra of samples oxidized at room temperature for 1 h; (a) without flow channel, (b) with FC900 and (c) with FC300. Source data are provided as a Source Data file.

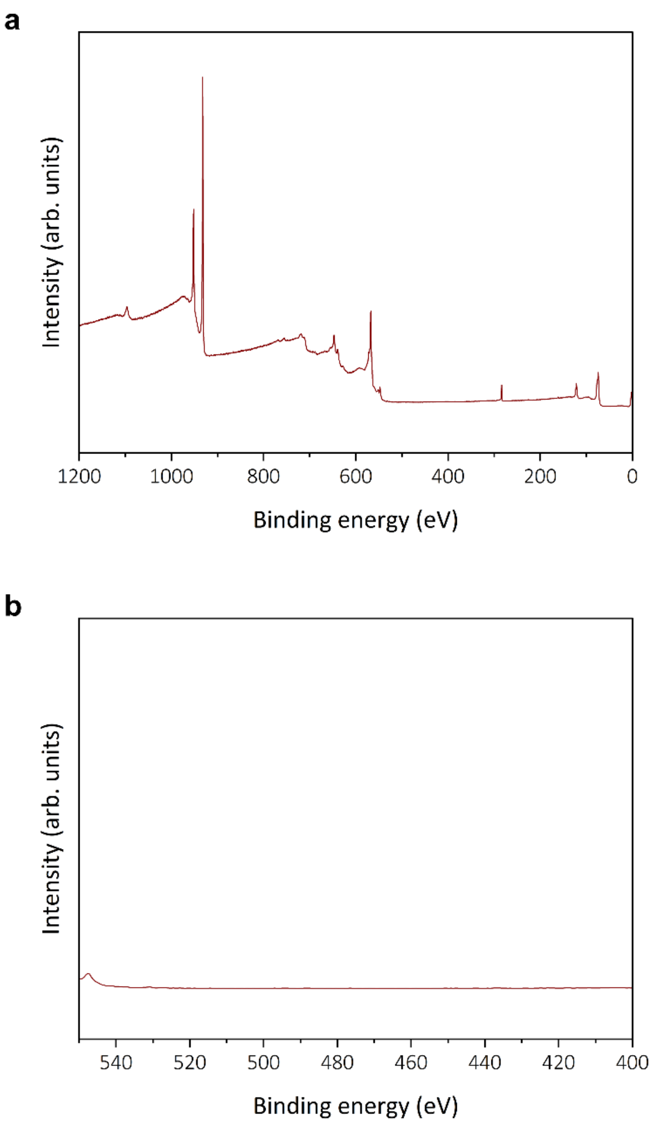

Figure S6. XPS wide spectra of pristine graphene sample. Source data are provided as a Source Data file.

Note S4. Estimation of mass transfer enhancement with increased O_3 flow velocity

Sherwood number shows the relation between convective mass transfer to diffusive mass transfer:

$$Sh = \frac{k_m L}{D}$$

where, k_m is the mass transfer coefficient, L is the characteristic length, D is the diffusivity.

For laminar flow between flat plates, the Sherwood number scales as [4]:

$$Sh = 1.85 Re^{1/3} Sc^{1/3}$$

and for tube:

$$Sh = 1.62 Re^{1/3} Sc^{1/3}$$

$$Re = \frac{\rho V L}{\mu}$$

$$Sc = \frac{\mu}{\rho D}$$

ρ is fluid density, μ is the dynamic viscosity, and V is the velocity. Therefore, the mass transfer coefficient scales with gas velocity as:

$$k_m \propto V^{1/3}$$

Table S2. The relative increase in mass transfer coefficients based on the average velocity of different flow configurations and corresponding C-O% content

**Condition**	**Velocity (cm/s)**	**C-O (%) from XPS**	**Relative k_m**
w/o Flow Channel	0.5	~ 4.1	1
FC900	15	~ 8.2	3.1 fold
FC300	45	~ 13	4.5 fold

For a first-order surface reaction, surface flux can be defined as:

$$J_{O_3} = k_m * C_{O_3}$$

If each reaction event yields a C–O bond, assuming that the bulk ozone concentration is constant, then the total C–O content after a fixed time is:

$$[C - O] \propto \int J_{O_3} dt \propto V^{1/3}$$

By comparing the XPS-measured C–O content with $V^{1/3}$, the relative increase in mass transfer coefficient can be confirmed.

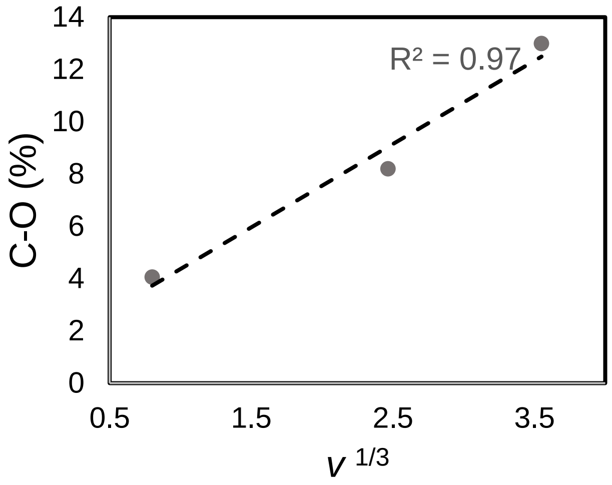

Figure S7. The change in C-O % content on graphene under different O_3 flow velocities. Source data are provided as a Source Data file.

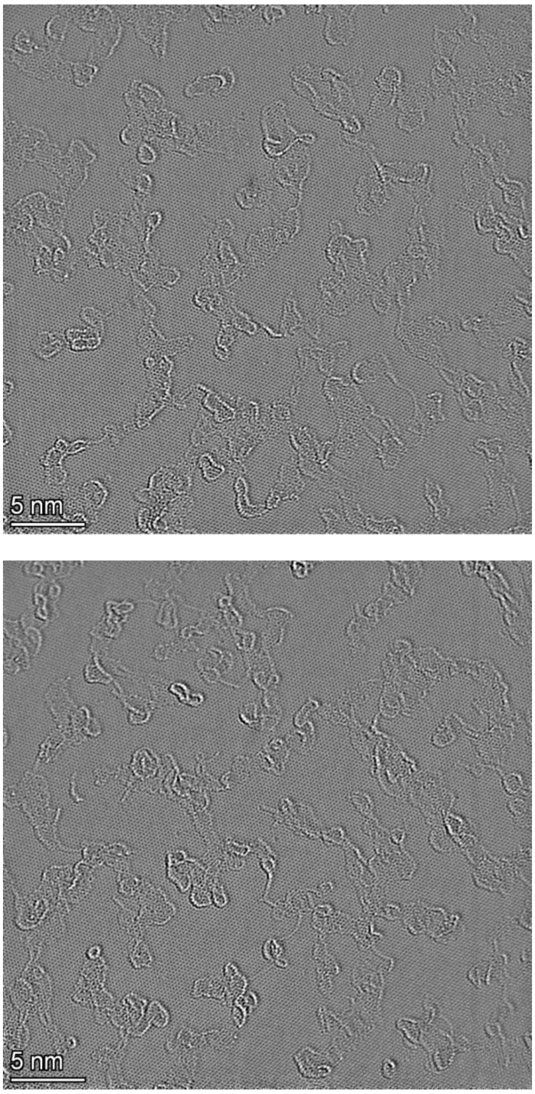

Figure S8. AC-HRTEM images of graphene sample oxidized at room temperature for 1 h, without a flow channel.

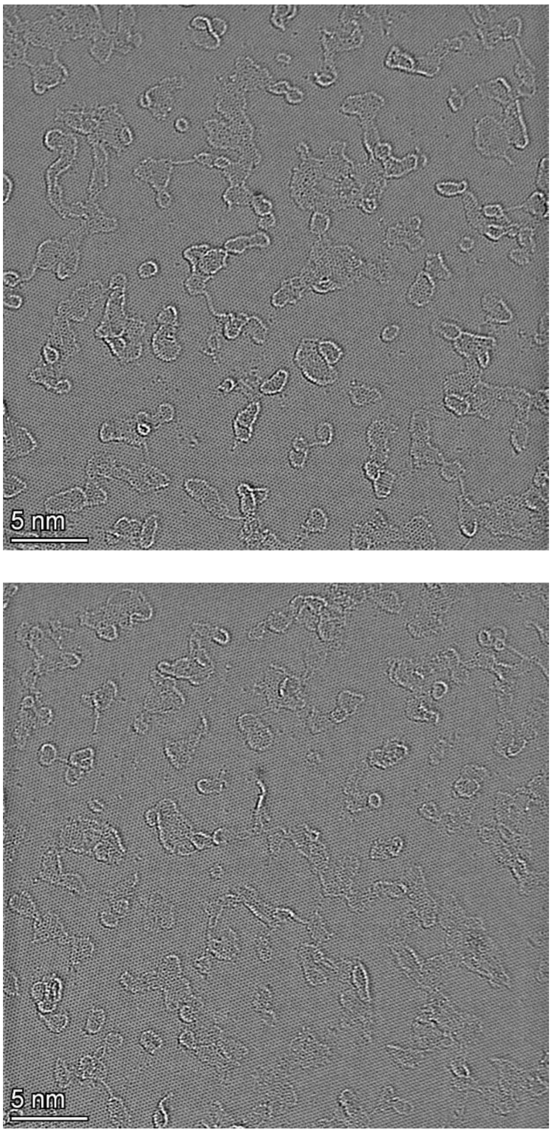

Figure S9. AC-HRTEM images of graphene sample oxidized at room temperature for 1 h, with FC900

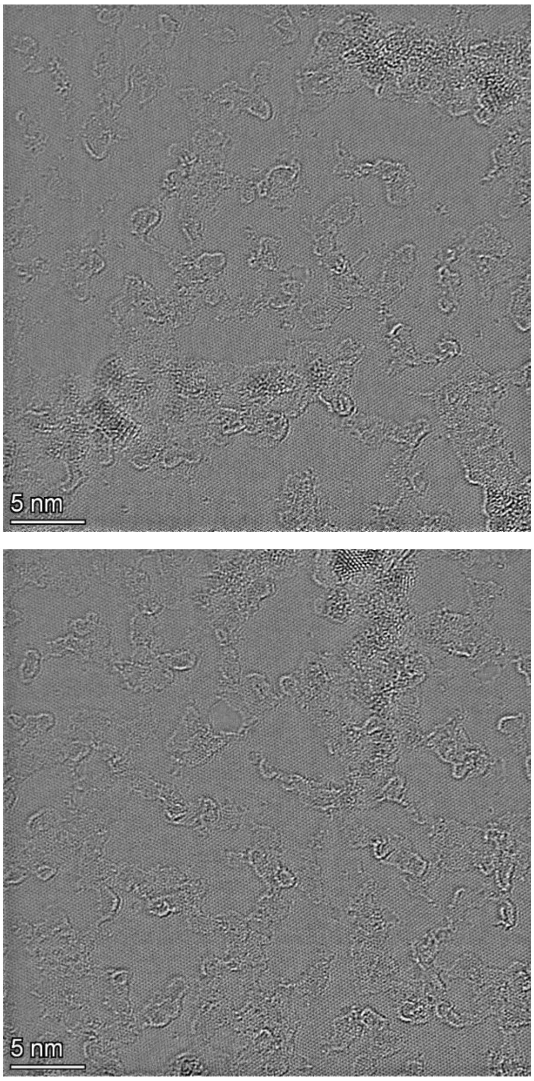

Figure S10. AC-HRTEM images of graphene sample oxidized at room temperature for 1 h, with FC300.

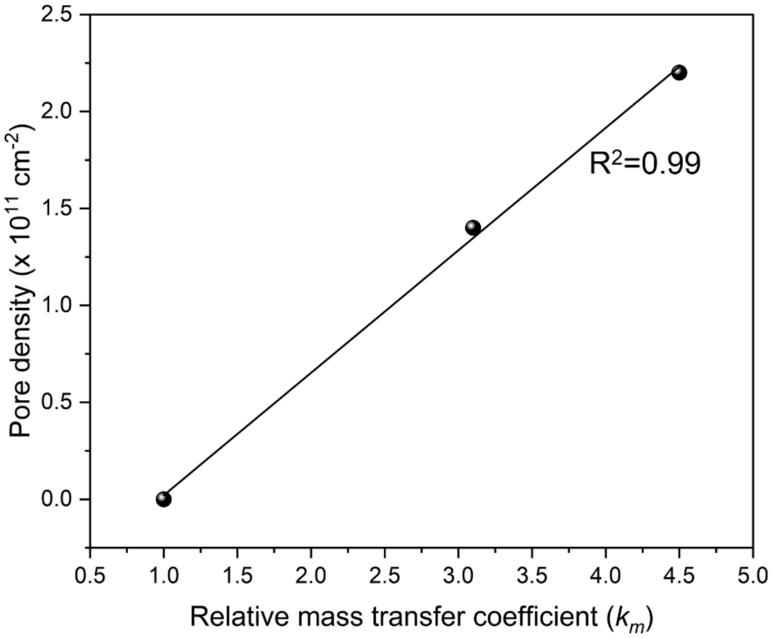

Figure S11. Change in the pore density with relative mass transfer coefficient (estimated based on $k_m \propto V^{1/3}$). Source data are provided as a Source Data file.

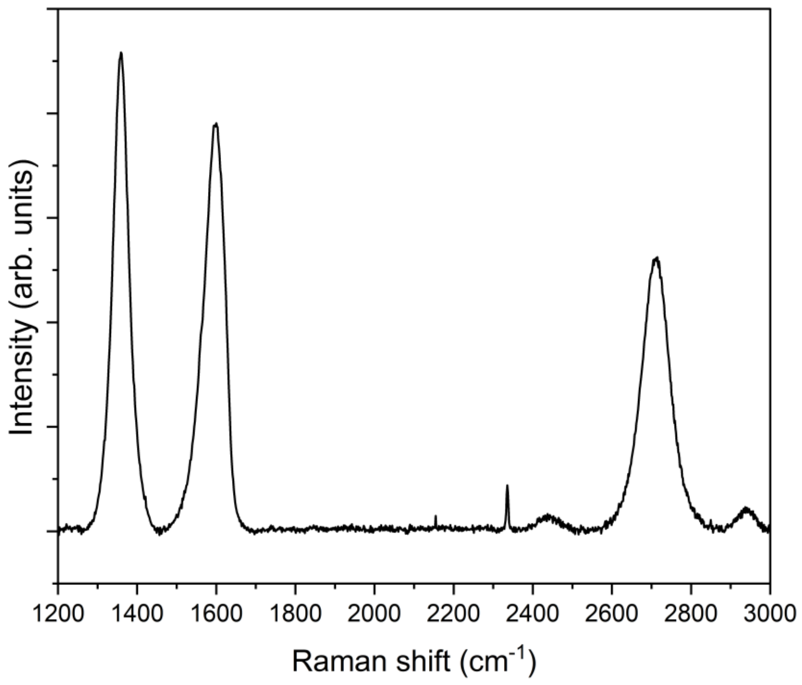

Figure S12. Raman spectra of graphene sample treated with ozone using FC900 for 1 h, followed by photonic gasification and post-ozone exposure for 2 h. Source data are provided as a Source Data file.

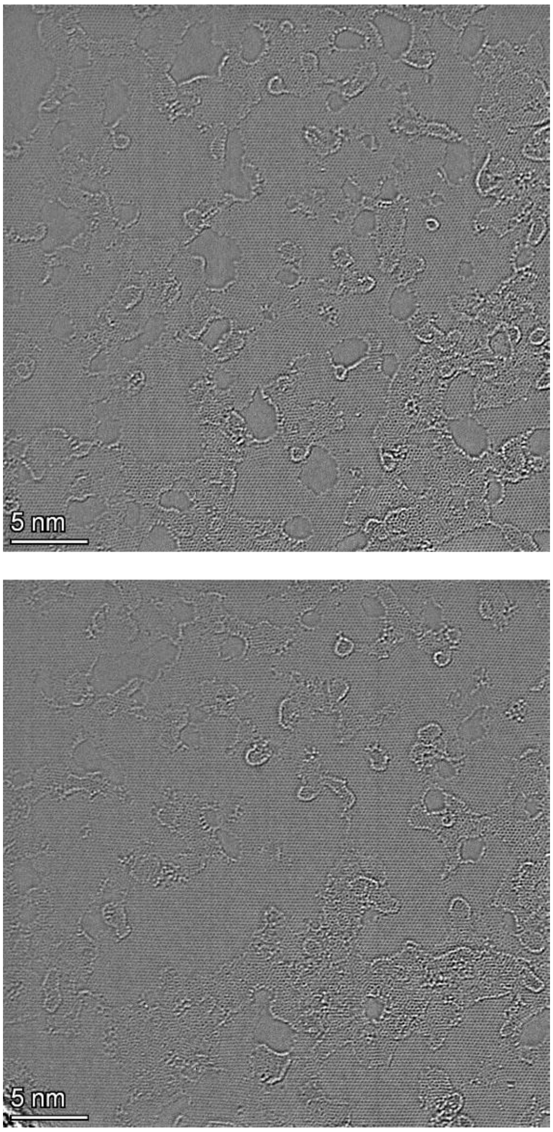

Figure S13. AC-HRTEM images of graphene sample oxidized at room temperature for 1 h, with FC900, after 15 min post expansion.

Note S5. Calculation of graphene layer permeance

Gas permeance of the porous graphene layer was determined by the resistance model, using the measured stack layer permeance values. The resistance (R) of the membrane through gas transport can be defined as:

$$R = \frac{1}{J.A}$$

A represents the actual membrane area, and J is the membrane flux.

The total resistance (R_T) of the graphene membrane is the sum of the resistances of layers ; graphene, PES and PTMSP supports.

$$R_{total} = R_{PES} + R_{graphene} + R_{PTMSP}$$

PES layer resistance can be neglected since it is extremely permeable, accordingly, graphene layer flux can be calculated by the following equation:

$$J_{graphene} = \frac{J_{total} * J_{PTMSP}}{J_{total} - J_{PTMSP}}$$

Table S3. CO_2 and N_2 gas permeances and CO_2/N_2 selectivities of graphene membranes (PTMSP CO_2 Permeance: 11125 ± 625 GPU, CO_2/N_2 Selectivity : 6.9 ± 0.6[5])

	**PTMSP/Gr/PES**			**Graphene Layer**		
	CO_2 (GPU)	N_2 (GPU)	CO_2/N_2	CO_2 (GPU)	N_2 (GPU)	CO_2/N_2
**w/o FC**	75	-	-	75.5	-	-
**w/o FC**	53	-	-	53.3	-	-
**w/o FC**	62	-	-	62.3	-	-
**FC900**	1407	79	17.6	1610	84	19.1
**FC900**	1395	88	15.8	1595	93	17
**FC900**	1610	100	16.1	1882	106	17.7
**FC900**	1490	88	17	1720	92.3	18.6
**FC900**	1210	80	15.3	1357	83	16.3
**FC900_2h**	1950	154	12.6	2364	171	13.8
**FC900_2h**	1620	114	14.1	1896	123	15.3
**FC300**	2345	128	18.3	2971	139	21.4
**FC300**	2691	178	16.6	4035	200	20.1
**FC300**	2557	213	12	3320	245	13.5
**FC900_5 min**	2419	155	15.6	3091	171	18
**FC900_5 min**	2700	180	15	3565	202	17.6
**FC900_5 min**	2972	188	16	4055	209	19.3
**FC900_5 min**	2750	200.8	13.7	3652	229	15.9
**FC900_5 min**	2870	215.8	13.3	3868	249	15.5
**FC900_15 min**	2883	171	16.8	3891	192	20.3
**FC900_15 min**	2872	159	18	3871	177	21.9
**FC900_15 min**	2740	161	17	3635	179	20.3
**FC900_30 min**	4150	518	8	6620	761	8.7
**FC900_30 min**	5410	688	7.9	10531	1193	8.8

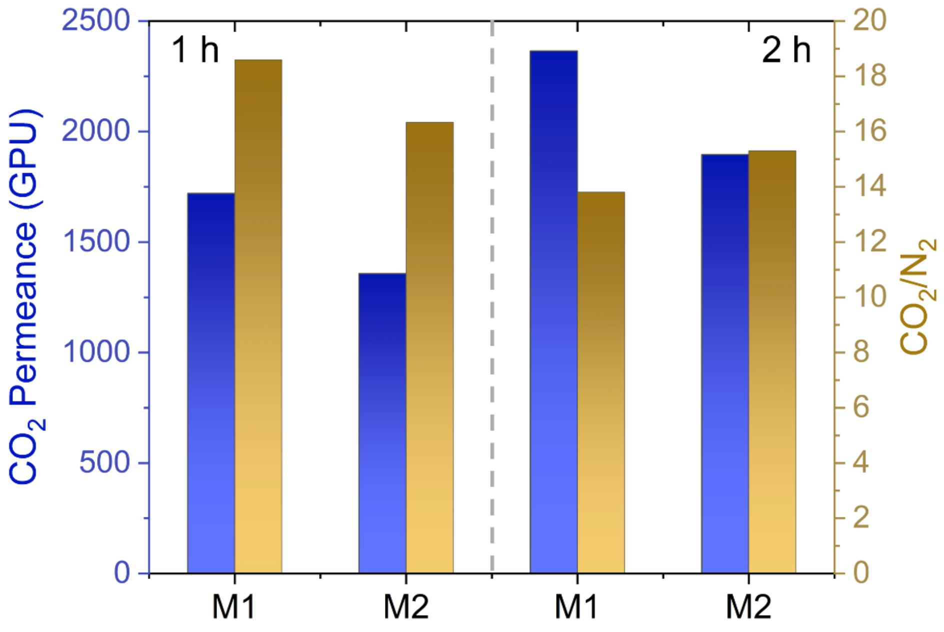

Figure S14. Gas separation performance of porous graphene fabricated using FC900, prepared using oxidation times of 1 and 2 h. Source data are provided as a Source Data file.

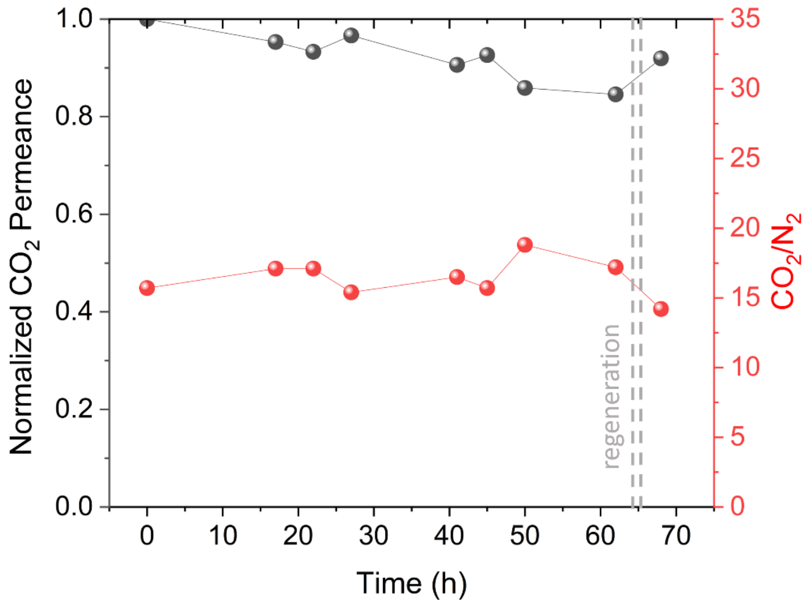

Figure S15. 70 h performance stability test of porous graphene membrane. Source data are provided as a Source Data file.

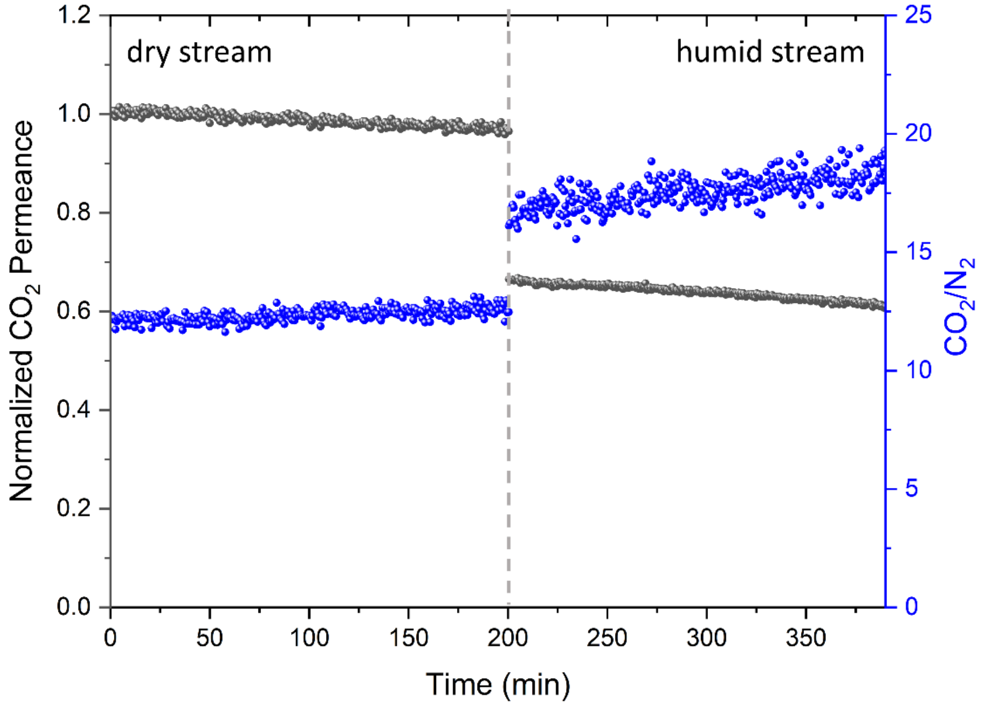

Figure S16. Gas mixture separation performance of porous graphene membrane prepared by 1 h oxidation in FC900 followed by 5 min 2nd cycle of ozone, under dry gas mixture (equimolar CO_2 and N_2) and humidified mixture (equimolar CO_2 and N_2 with 3% water vapor) feed. Initial performance with dry stream taken as reference. Source data are provided as a Source Data file.

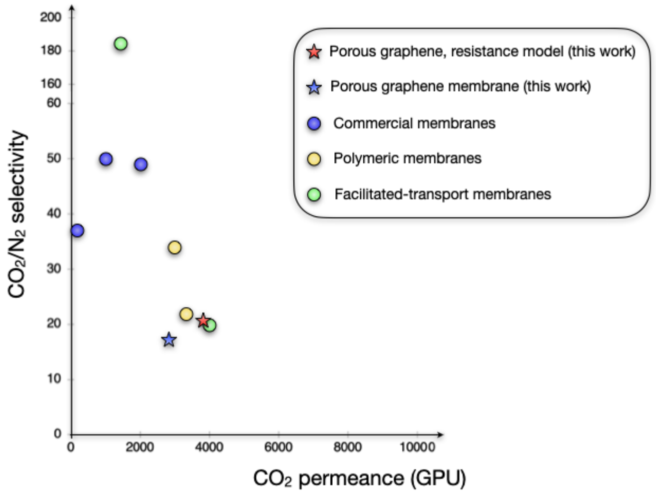

Figure S17. Gas separation performances of state-of-art and commercial membranes for CO_2/N_2 separation

Table S4. Comparison of carbon capture performance of porous graphene membrane.

Membrane Type	Note	CO_2 permeance (GPU)	CO_2/N_2 selectivity (separation factor)	Reference
Porous Single Layer Graphene	FC300	2691	16.6	This work
	FC300, resistance model	4035	21	
Commercial membranes	(Gen 1) Polaris ®	1000	50	[6]
	(Gen 2) Polaris ®	2000	49	[7]
	Prism	161	37	[8]
Polymeric membranes	Pebax2533/PEG-b-PPFPA	3330	22	[9]
	PEG/NH2-MIL-53	3000	34	[10]
Facilitated transport membranes	Ionic liquid on graphene	4000	20	[11]
	Amine-incorporated polymer	1450	185	[12]

References

(1) Ferrari, A. C.; Basko, D. M. Raman Spectroscopy as a Versatile Tool for Studying the Properties of Graphene. *Nat. Nanotechnol.* **2013**, *8* (4), 235–246. https://doi.org/10.1038/nnano.2013.46.

(2) Cançado, L. G.; Jorio, A.; Ferreira, E. H. M.; Stavale, F.; Achete, C. A.; Capaz, R. B.; Moutinho, M. V. O.; Lombardo, A.; Kulmala, T. S.; Ferrari, A. C. Quantifying Defects in Graphene via Raman Spectroscopy at Different Excitation Energies. *Nano Lett.* **2011**, *11* (8), 3190–3196. https://doi.org/10.1021/nl201432g.

(3) He, G.; Huang, S.; Villalobos, L. F.; Zhao, J.; Mensi, M.; Oveisi, E.; Rezaei, M.; Agrawal, K. V. High-Permeance Polymer-Functionalized Single-Layer Graphene Membranes That Surpass the Postcombustion Carbon Capture Target. *Energy Environ. Sci.* **2019**, *12* (11), 3305–3312. https://doi.org/10.1039/c9ee01238a.

(4) Wang, J.; Dlamini, D. S.; Mishra, A. K.; Pendergast, M. T. M.; Wong, M. C. Y.; Mamba, B. B.; Freger, V.; Verliefde, A. R. D.; Hoek, E. M. V. A Critical Review of Transport through Osmotic Membranes. *J. Membr. Sci.* **2014**, *454*, 516–537. https://doi.org/10.1016/j.memsci.2013.12.034.

(5) Hao, J.; Mieczyslaw Gebolis, P.; Marcin Gach, P.; Chevalier, M.; Sébastien Bondaz, L.; Kocaman, C.; Hsu, K.-J.; Bhorkar, K.; Babu, D.; Agrawal, K. V. Scalable Synthesis of CO2-Selective Porous Single-Layer Graphene Membranes. *Nat. Chem. Eng.* **2025**, No. 2, 241–251.

(6) Merkel, T. C.; Lin, H.; Wei, X.; Baker, R. Power Plant Post-Combustion Carbon Dioxide Capture: An Opportunity for Membranes. *J. Membr. Sci.* **2010**, *359* (1–2), 126–139. https://doi.org/10.1016/j.memsci.2009.10.041.

(7) White, L. S.; Amo, K. D.; Wu, T.; Merkel, T. C. Extended Field Trials of Polaris Sweep Modules for Carbon Capture. *J. Membr. Sci.* **2017**, *542*, 217–225. https://doi.org/10.1016/j.memsci.2017.08.017.

(8) Janusz-Cygan, A.; Jaschik, J.; Wojdyła, A.; Tańczyk, M. The Separative Performance of Modules with Polymeric Membranes for a Hybrid Adsorptive/Membrane Process of CO2 Capture from Flue Gas. *Membranes* **2020**, *10* (11), 309. https://doi.org/10.3390/membranes10110309.

(9) Scofield, J. M. P.; Gurr, P. A.; Kim, J.; Fu, Q.; Kentish, S. E.; Qiao, G. G. Development of Novel Fluorinated Additives for High Performance CO2 Separation Thin-Film Composite

Membranes. *J. Membr. Sci.* **2016**, *499*, 191–200. https://doi.org/10.1016/j.memsci.2015.10.035.

(10) Xie, K.; Fu, Q.; Xu, C.; Lu, H.; Zhao, Q.; Curtain, R.; Gu, D.; Webley, P. A.; Qiao, G. G. Continuous Assembly of a Polymer on a Metal–Organic Framework (CAP on MOF): A 30 Nm Thick Polymeric Gas Separation Membrane. *Energy Environ. Sci.* **2018**, *11* (3), 544–550. https://doi.org/10.1039/C7EE02820B.

(11) Guo, W.; Mahurin, S. M.; Unocic, R. R.; Luo, H.; Dai, S. Broadening the Gas Separation Utility of Monolayer Nanoporous Graphene Membranes by an Ionic Liquid Gating. *Nano Lett.* **2020**, *20* (11), 7995–8000. https://doi.org/10.1021/acs.nanolett.0c02860.

(12) Chen, K. K.; Salim, W.; Han, Y.; Wu, D.; Ho, W. S. W. Fabrication and Scale-up of Multi-Leaf Spiral-Wound Membrane Modules for CO2 Capture from Flue Gas. *J. Membr. Sci.* **2020**, *595*, 117504. https://doi.org/10.1016/j.memsci.2019.117504.
